# Fully-Automated Identification of Imaging Biomarkers for Post-Operative Cerebellar Mutism Syndrome Using Longitudinal Paediatric MRI

**DOI:** 10.1007/s12021-019-09427-w

**Published:** 2019-06-28

**Authors:** Michaela Spiteri, Jean-Yves Guillemaut, David Windridge, Shivaram Avula, Ram Kumar, Emma Lewis

**Affiliations:** 1 Centre for Vision, Speech and Signal Processing (CVSSP), University of Surrey, Guildford, GU27XH UK; 2 Alder Hey Children’s NHS Trust, E Prescot Rd, Liverpool, L14 5AB UK

**Keywords:** MRI, POPCMS, Posterior fossa, Longitudinal, Machine learning

## Abstract

Post-operative cerebellar mutism syndrome (POPCMS) in children is a post- surgical complication which occurs following the resection of tumors within the brain stem and cerebellum. High resolution brain magnetic resonance (MR) images acquired at multiple time points across a patient’s treatment allow the quantification of localized changes caused by the progression of this syndrome. However, MR images are not necessarily acquired at regular intervals throughout treatment and are often not volumetric. This restricts the analysis to 2D space and causes difficulty in intra- and inter-subject comparison. To address these challenges, we have developed an automated image processing and analysis pipeline. Multi-slice 2D MR image slices are interpolated in space and time to produce a 4D volumetric MR image dataset providing a longitudinal representation of the cerebellum and brain stem at specific time points across treatment. The deformations within the brain over time are represented using a novel metric known as the Jacobian of deformations determinant. This metric, together with the changing grey-level intensity of areas within the brain over time, are analyzed using machine learning techniques in order to identify biomarkers that correspond with the development of POPCMS following tumor resection. This study makes use of a fully automated approach which is not hypothesis-driven. As a result, we were able to automatically detect six potential biomarkers that are related to the development of POPCMS following tumor resection in the posterior fossa.

## Introduction and Background

Post-operative cerebellar mutism syndrome (POPCMS) is a post-surgical complication whose exact underlying pathophysiological mechanism is unclear. However, it is widely considered to involve disruption of the proximal efferent cerebellar pathways (pECP) that connect the cerebellum to the forebrain (Kirk et al. 1995; Lavezzi et al. 2009; Patay et al. 2014; Avula et al. 2015a). In order to enable better diagnosis and management of children with this disorder, as well as increase understanding of the underlying mechanisms, it is important to identify imaging biomarkers that are associated with it.

The aim of this study is to identify biomarkers for POPCMS following tumor resection surgery in the posterior fossa, and to do so through a fully automated process which is not hypothesis drive. In order to do so, changes in grey-level intensity within distinct lobules in the brain stem and the cerebellum were analyzed longitudinally. Morphological change within these lobules following tumor resection, which is also thought to contribute to the patient’s recovery after surgery, was also analyzed in this way. The fully-automated pipeline presented in this paper may be applied to similar longitudinal analysis problems.

Our main approach was to ensure that the entire cerebellum and brain stem were assessed longitudinally in 3D space. The complete pipeline is fully auto- mated, and features were not chosen manually. This ensured that the study was not biased to clinical hypotheses. The MR image dataset posed some difficulties. The post-operative MR images were acquired in the T2 modality, which is well-suited to imaging hypertrophy, however they were acquired in Spiral MR and were not volumetric images. Furthermore, the images were not acquired at regular intervals throughout each subject’s treatment.

The analysis of brain tumors in adults, and the extraction of imaging biomarkers from MR images, have both been the subject of extensive research over the past decade (Levman and Takahashi 2016; Bauer et al. 2013). A substantial number of longitudinal studies in the field of brain MRI consist of the identification of biomarkers in MR images, determined by neuroradiological visual assessment, followed by a quantitative statistical analysis of these biomarkers, observing the degree of correlation be- tween the occurrence of these biomarkers and the occurrence of a measurable outcome. An example of this is demonstrated by Avula et al. (2015), in which abnormalities in the proximal efferent cerebellar pathway (pECP) and the cerebellar vermis, as seen on diffusion weighted MRI, are evaluated in relation to the occurrence of PFS (Avula et al. 2015b). Patay et al. (2014) also analyzed the disruption of cerebellar pathways longitudinally, specifically those that make up the pECP, and the occurrence of PFS. In another longitudinal study of a similar scope Kupeli et al. (2011) performed a multivariate analysis on the correlation between PFS and mutism with other factors such as socio-economic background, location of tumor and histopathological diagnosis (Kupeli et al. 2011). These quantitative studies are based heavily on human assessment which may be prone to intra- and inter-observer variability. Furthermore, such assessments are time-consuming, tedious and may be difficult to reproduce.

An automatic assessment of a longitudinal dataset does not only improve the efficiency of analyzing data, but, together with the use of machine learning techniques, may also improve accuracy and provide information on structures that are not always visible to the naked eye. The application of automatic image analysis and machine learning in longitudinal pediatric MRI datasets has not been explored extensively, however there exist several studies of similar scopes applied to the longitudinal analysis of adult brain MRI (Giedd et al. 1999; Simpson et al. 2011).

There exist many applications of machine learning to segment and identify brain lesions, for example the use of a decision forest to classify pixels automatically into tissue types in order to identify tumor volume (Meier et al. 2016). A number of other techniques were presented at MICCAI BRATS challenge 2016, which was aimed at automatically segmenting tumors from longitudinal datasets (consisting of two consecutive MR images) in order to analyze volumetric changes over time. The majority of segmentation techniques presented in this challenge made use of a registration step, followed by different variations of convolutional neural networks (CNNs) (Dera et al. 2016; Kamnitsas et al. 2017; Kamnitsas et al. 2016; Piedra et al. 2016; Zeng et al. 2016), however deformations between two consecutive images in the dataset were not analyzed, providing no information regarding morphological change within the lesion or the rest of the brain over time.

Although the methods presented in the MICCAI BRATS challenge are excellent models for brain lesion segmentation, they do not present methods which are specific to solving the problem of sparsely sampled longitudinal datasets. Furthermore the registration step was carried out with the sole purpose of aiding the segmentation process, and the resultant deformations fields were not analyzed, providing no information regarding morphological change over time (Piedra et al. 2016; Zeng et al. 2016). Other studies include the application of a bee colony algorithm to classify brain MR into normal and abnormal cases by Zhang and Wu (Zhang and Wu 2011), which was later adapted to make use of a kernelized SVM to perform this classification (Zhang and Wu 2012), and the application of deep learning to identify MR images with abnormalities (Singh et al. 2012). In both cases the techniques were applied to non-longitudinal data, and therefore the techniques presented were not directly applicable to our dataset. The pipeline presented in this study makes use of both image processing principles as well as machine learning principles. In this study, unlike the previous study in (Spiteri et al. 2015), image segmentation was applied to extract the cerebellum and brain stem from the MR images. In order to allow a longitudinal analysis of intra-subject and inter-subject variability, the spiral MR slices were resliced in 3D space. Following this, registration techniques were applied in order to align the images to a common atlas in order to extrapolate the missing scans across the subject’s treatment. Feature extraction was performed in a blinded and automated fashion, in a manner which is not hypothesis driven. Features were obtained directly from the MR images as well as from the deformation field obtained as a result of registering the MR images to a cerebellum template. Feature selection techniques were applied to these features in order to identify the optimal feature set, referred to as biomarkers. A kernelized classification model was then applied to these biomarkers in order to demonstrate classification accuracy when using these biomarkers to classify subjects into POPCMS and non-POPCMS groups.

## Materials and Methods

### Participants and Imaging Protocol

The dataset was compiled from 40 patients who were treated for various histological types of posterior fossa tumors at Alder Hey Children’s Hospital between 2007 and 2013. The patients were aged between 8 months and 18 years of age (at surgery), seven of whom were diagnosed with POPCMS, the diagnosis of which was made by a neurologist following the review of clinical documentation. The methodology of the study did not fulfill the criteria for research ethics approval requirement and was given institutional approval by the Director of Research at Alder Hey Children’s Hospital.

Follow up MR images during one-year post-surgery were reviewed and up to five MR images were acquired longitudinally for each patient across their treatment. A small subset of these datasets included intra-operative MR images. Table 1 describes the MR dataset acquired for each patient, showing the number of days after surgery when an MR image was acquired. The same annotation as our previous study was used (Spiteri et al. 2015), such that negative numbers indicate a pre- operative MR image. Pre-operative scans acquired on the day of surgery, or intra-operative scans, are indicated as 0. Most patients were followed-up every 3 months, whilst others with potentially malignant tumors were followed-up more frequently. A mean of 4 ± 1 MR images were acquired for each patient with a mean time interval of 109 ± 62 days between each image acquisition. The POPCMS column refers to the clinical diagnosis made by a neurologist, in which a 1 indicates presence of the syndrome, and a 0 indicates otherwise.

**Table 1:** Patient longitudinal dataset

Patient	Image acquisition: days after surgery						POPCMS
1	0	1	110	194	/	/	0
2	0	1	118	202	286	398	0
3	−5	120	204	288	400	/	0
4	−33	1	59	94	225	395	0
5	0	1	90	202	314	488	1
6	−1	1	98	231	413	/	0
7	−3	1	93	114	116	162	0
8	−2	1	48	100	280	651	0
9	−7	1	147	287	511	/	0
10	−1	1	390	/	/	/	0
11	26	32	138	278	474	/	0
12	0	0	175	287	403	/	0
13	0	2	48	/	/	/	0
14	−11	0	143	318	437	/	0
15	−2	1	25	118	278	481	0
16	−2	4	103	551	/	/	0
17	−4	1	176	260	372	/	1
18	−190	0	189	273	357	/	0
19	−1	2	96	193	216	334	0
20	28	1	215	299	355	/	0
21	−2	1	117	201	356	/	0
22	−4	1	147	343	/	/	0
23	−1	3	18	21	165	228	0
24	−4	0	76	163	191	251	0
25	−1	0	55	136	257	440	0
26	−4	0	50	169	309	486	0
27	−6	0	89	285	/	/	0
28	−1	0	110	446	/	/	0
29	0	0	124	288	481	/	1
30	−8	1	2	97	181	321	1
31	−22	0	76	84	242	329	0
32	−52	0	14	14	126	126	0
33	0	0	108	255	445	/	0
34	−1	0	4	65	203	297	1
35	−1	0	5	62	165	265	0
36	−6	0	19	394	201	322	0
37	−2	0	173	509	/	/	0
38	−1	0	53	174	218	319	1
39	0	0	178	273	424	/	1
40	−1	0	28	82	166	189	0

T2 weighted sequences from the pre-, intra- and post-operative scan were used to evaluate for signal change within the posterior fossa and the following parameters were used: TR = 4485 ms, TE = 11 ms, slice thickness = 6 mm, number of slices = 20, time-step = 4.49 s. The pre-operative and post-operative MR images were acquired using 1.5 T or 3 T MRI. Intra-operative MR images were acquired using 3 T MRI. The T2 sequence was used due to its ability to identify cerebrospinal fluid, blood and edema as increased grey-level intensity and similarity of the images and parameters on 1.5 and 3 T scans.

Volumetric T2 weighted imaging is not routinely used in pediatric brain tumor surveillance imaging, as it is time consuming and prone to movement associated artefacts. Volumetric T1 weighted imaging is sometimes performed but this is less sensitive in identifying edema or gliosis that represents pathological change. Instead, axial T2 spin echo (SE) or turbo spin echo (TSE) sequences were used to evaluate for T2 related changes that can demonstrate residual/recurrent tumor and changes related to therapy including surgery. These T2 MR images were acquired in Spiral MRI which captures the k-space through a spiral trajectory. This method of acquisition is fast and results in high in-plane spatial resolution, giving improved resolution of the brain parenchyma including small structures such as the ION (Tan 2009; Delattre et al. 2010).

### Image Processing

#### Overview

In order to perform an automated analysis of MR images, it is preferable to represent the images in a uniform format, such that the size and image intensity range of the MR images is consistent across all patients, and such that the same number of post-operative MR images is analyzed for each patient. Representing the images in a uniform format enabled more general processes to be applied to the dataset without having to make exceptions for the inconsistencies in the data. Image registration is used to spatially align the images to a common atlas. In this process, voxels in the MR images are mapped to voxels of a synthesized cerebellum and brain stem template. Prior to registering the MR images, it is useful to perform a segmentation step, by which only relevant parts of the MR images are retained, whilst the rest are discarded. This important step serves the purpose of reducing the search space for the registration step and for the following machine learning processes.

**Fig. 1:**
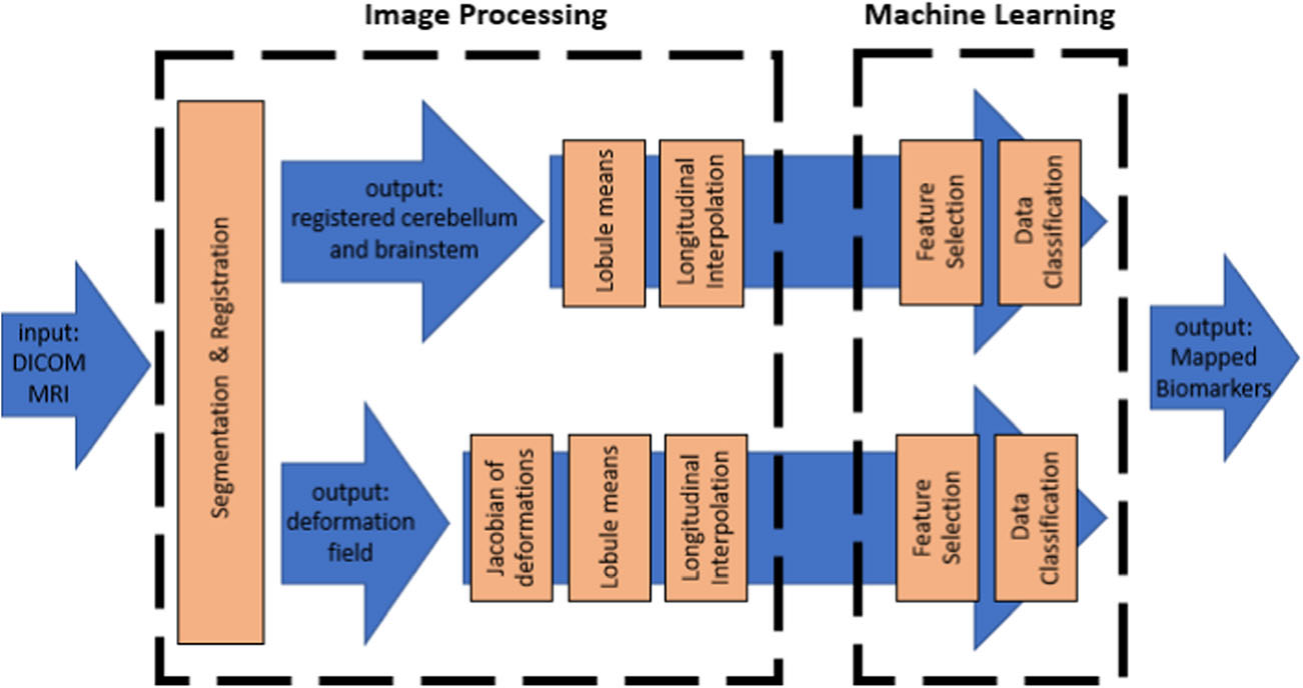
Block diagram of automated analysis pipeline composed of an image processing block and a machine learning block

The fully-automated analysis pipeline is shown in Fig. 1. The pipeline is split into two main blocks: an image processing block and a machine learning block.

#### Cerebellum Segmentation and Registration

Segmentation was performed on the brain in order to analyze the cerebellum in isolation. The choice of a template for use in segmenting small structures within the posterior fossa and enabling intra/inter-subject registration poses several difficulties:

The difference in the volumetric T1 and spiral T2 imaging modalities gives rise to difficulties in matching similar regions within the brain.

Most templates, such as the MNI whole-brain template, lack definition in small structures within the pECP such as the deep cerebellar nuclei, which are highly relevant to the development of POPCMS (Avula et al. 2015b).

The most frequently used template for brain image analysis is the ICBM152 template which defines the commonly known MNI space (Fonov et al. 2011; Fonov et al. 2009). This template was constructed by obtaining the mean of 152 individual brains after these brains were normalized and corrected for scaling, translation and rotation. However, this template is not sufficient for cerebellar structures as it provides minimal contrast in this area of the brain. For this reason, a high-resolution atlas template of the cerebellum was used, named SUIT. This template was constructed using the images of 20 young healthy brains. SUIT is spatially unbiased and the locations of structures is matched to the expected location of these structures in MNI space, however it provides better detail of cerebellar structures by using a non-linear atlas-generation algorithm. This allows a more accurate inter-subject alignment including lobules and deep cerebellar nuclei (Diedrichsen 2006a; Diedrichsen et al. 2009; Diedrichsen et al. 2011; Diedrichsen and Zotow n.d.).

Both affine and non-linear spatial normalization of the brain to the NMI tem- plate result in a resliced registration error in alignment for the center of mass of primary fissures and intra-biventer fissures within the brain. The distance between these landmarks on two individual subjects is around 4 mm on average which is due to the large variability in the MNI’s template folding pattern of the neo-cortex. The SUIT template preserves the anatomical detail of the cerebellum to a much greater degree than the MNI template of the whole brain. The post-registration overlap of the fissures is improved, reducing spatial variance of the centers of mass by 66%. The overlap of the deep cerebellar nuclei is also superior to that of MNI space, which is of specific relevance to this project as it provides accurate alignment of the inferior olivary nuclei (Diedrichsen et al. 2009).

The cerebellum segmentation process is split into 5 steps:

- Isolation: The entire un-registered brain MR image is segmented into white matter, grey matter and cerebrospinal fluid by assigning a probability to each voxel. The volume is then cropped to only include the voxels which are most likely to form part of the cerebellum and brain stem. The SUIT toolbox includes an isolation algorithm that segments the brain based on tissue type based on a diffeomorphic registration with Gauss-Newton optimization. This algorithm crops the part of the brain which includes the infra-tentorial structures and eliminates the rest. It does this by computing the posterior probability of each voxel belonging to the posterior fossa, based on the tissue-type within the voxel and the proximity of the voxel to cortical matter. The probability map obtained is then thresholded at *p* > 0.5 in order to determine which pixels are more probably part of the brain stem and the cerebellum (Diedrichsen et al. 2009).
- Registration: The voxel intensities are first normalized such that the grey-level intensity range of each query MR image matches that of the atlas template used in the SUIT algorithm. The registration process is composed of an affine registration step and a non-rigid registration step. The full brain MR image has been cropped in the previous step, and voxels have been labeled a priori according to tissue type, providing a basis for the first step of the registration, which is a 12 degrees-of-freedom affine registration. Three of the parameters are derived from the 3D translation, whilst the other 9 are derived from a matrix combining rotation, scaling and shearing. These parameters are optimized by least-squares minimization. An affine transformation matrix is then extracted from the generalized affine transformation (Ashburner and Friston 1997). The non-rigid registration is described by three properties: the deformation field - which is modelled by linear combinations of low frequency components of the three-dimensional discrete cosine transform (DCT), the cost function regularization - which is based on the membrane energy of the deformations, and optimization function - which is derived from the partial derivatives of the basis functions - which optimizes the overlap of the center of gravity of the moving image and the fixed image (template) (Diedrichsen 2006a; Diedrichsen et al. 2009; Diedrichsen et al. 2011; Diedrichsen and Zotow n.d.).
- Reslicing: The deformation field obtained in the previous step is used to reslice the image back into its original image space. This is done by applying the inverted deformation field to the masked image obtained in the previous step. The mask is applied to isolate the cerebellum and brain stem from the rest of the brain. The application of the mask avoids contaminations from adjacent visual cortex voxels when the final segment is smoothed. The size and shape of the brain may differ greatly from one subject to another. In order to accurately compare one subject to another, the segmented posterior fossa is deformed to obtain the best correspondence to the template. In order to facilitate the matching process, normalization is carried out. The SUIT toolbox provides superior normalization to that of the MNI whole-brain template. This is because it provides better alignment of the individual fissures, reducing discrepancies by 60% and improving the overlap of the deep cerebellar nuclei. The normalization algorithm calculates the non-linear deformation map between the segmented posterior fossa and the SUIT template. This is done using the cosine-basis function (Ashburner and Friston 1999). The algorithm outputs are a deformation field and a resliced version of the masked, normalized image.
- Thresholding: Although the original image is masked to eliminate voxels of a low-likelihood, the resultant image is a probability map, which assigns a probability to each voxel indicating its likelihood of forming part of cerebellar and brain stem tissue. A threshold is carried out to eliminate voxels which are of a very low probability.
- Longitudinal Registration: All the brain MR images in the longitudinal dataset were then deformed to the template space by performing the registration step once more. This resulted in a longitudinal dataset of images that were all in the same spatiotemporal space, allowing the quantification of changes in the brain over time. The registration process, which involved an intensity normalization step, ensured that the voxel intensities were normalized across each patient’s longitudinal dataset, eliminating errors due to different imaging modalities. The registration step provided two outputs: a deformed (or moving) image which is spatially aligned to the reference image (or fixed image) and a deformation field which represents the deformation between the deformed image and the reference image.

#### Cerebellar Lobule Grey-Level Intensity Means

The SUIT atlas contains transverse MR image slices of the cerebellum, the voxels of which are labelled according to the cerebellar lobule they belong to. The atlas is symmetrical and corresponding lobules on the left and right side of the cerebellum are given the same grey-level intensity value, giving rise to 35 distinct grey-level intensities across the entire atlas. It was desired to acquire information on whether a potential biomarker is located on the left or the right side of the cerebellum. For this reason, the atlas was halved and the lobules on the left side of the cerebellum were given new grey-level intensity values in order to distinguish them from their right counterparts. Following this, each registered MR image was masked with this atlas and the mean grey-level intensity value of voxels falling within the same lobule was calculated. This resulted in 70 distinct mean grey-level intensity values for each registered image.

#### Longitudinal Interpolation of Cerebellar Lobule Means

In order to compare the deformations and intensities within the brain throughout the period over which the subject was monitored post-operatively, it was necessary to interpolate each of the 70 cerebellar lobule means over this period. The entire dataset was analyzed over a period of 7 months, due to the limited number of MR scans available after this date. Due to the different pathophysiological profile of each subject, MR scans were not acquired at equal intervals for each patient. For this reason, subjects had missing MR scans (and therefore missing cerebellar lobule means) at different points throughout the 7-months period. It was therefore necessary to interpolate the cerebellar lobule means longitudinally in order to infill the gaps resulting from the missing scans. The interpolation method used was trilinear interpolation. In order to do this, the patient must have had at least 2 MR scans acquired over the 7-months period. The interpolation process resulted in 490 (70 cerebellar lobule means × 7 months) data points for each subject.

#### Determinant of Jacobian of Deformations

In addition to analyzing the change in intensity of regions of the brain over time, it is important to also analyze the significance of the deformations in different regions of the brain longitudinally. In order to do this, the determinant of the Jacobian of deformations was calculated. The Jacobian matrix is a measure of the expansion or shrinkage of voxels within the brain, based on the deformation field vectors. In order to produce the Jacobian matrix, the first order partial derivatives of each deformation vector for each voxel within the matrix was computed (Hua et al. 2009). This is defined by Eq. 1, which defines the Jacobian matrix for voxels in the coordinate space *x, y, z* deformed to the new coordinate space *u, v, w*. The Jacobian matrix results in a matrix of numbers the size of the original image for which the deformation field is computed. A Jacobian matrix element value of 1 indicates no change for that particular voxel; a value below 1 indicates shrink- age whilst a value above 1 indicates expansion for that particular voxel. The cerebellar lobule means calculation and longitudinal interpolation, which were applied to the MR image voxels, were also applied to the Jacobian of deformation matrices, resulting in an additional 490 data points for each subject.

1 $$ \frac{\partial \left(x,y,z\right)}{\partial \left(u,v,w\right)}=\left|\begin{array}{ccc}\frac{\partial x}{\partial u}& \frac{\partial x}{\partial v}& \frac{\partial x}{\partial w}\\ {}\frac{\partial y}{\partial u}& \frac{\partial y}{\partial v}& \frac{\partial y}{\partial w}\\ {}\frac{\partial z}{\partial u\ }& \frac{\partial z}{\partial v\ }& \frac{\partial z}{\partial w\ }\end{array}\right| $$

### Machine Learning

This section sets out the use of machine learning techniques to automate the identification of potential biomarkers from a longitudinal dataset of MR images. The machine learning section of this study was based upon the approached presented in (Spiteri et al. 2015), however the feature selection techniques were implemented twice, once to analyze the cerebellar lobule grey-level intensity means and a second time to analyze the cerebellar lobule Jacobian of deformation means, due to the large size of the feature set in comparison to the number of subjects. Following feature selection, a kernelized support vector machine was applied to the highest scoring features, in order to validate their discriminative abilities.

#### Feature Selection

A feature selection method was applied to the cerebellar lobule means across a 7-months period in the aim of identifying both a location within the brain and a time window which are relevant to the development of POPCMS.

The technique used to identify these salient features is known as recursive feature elimination (RFE). Similarly to previous studies by Spiteri et al. (2015), the feature selection process has been applied as a filter method and is similar to the sequential feature selection algorithm (SFS) described in (Spiteri et al. 2015; Avula et al. 2016). The main difference between RFE and SFS is that instead of sequentially adding features to a subset, the algorithm starts off with the full set of features and iteratively removes the weakest feature (or features) at each iteration. This process is repeated until the specified number of features is reached. In this case, the optimal number of features is not known in advance, therefore stratified cross-validation was used to score different feature subsets and selecting the best coring collection of features. This adaptation is known as recursive feature elimination with cross-validation (RFECV).

This algorithm forms part of the open-source Scikit-Learn Machine Learning Package API for Python. The documentation for the scikit-learn API can be found in (Recursive Feature Elimination 2018).

The feature selection process was applied twice: once to the cerebellar lobule means of the grey-level intensities, and once to the cerebellar lobule means of the Jacobian of deformations. For each application a 6-fold stratified cross-validation was carried out, with the data equally split into six separate subsets with an equal representation of POPCMS subjects in each group; one of the subsets is reserved for testing, whilst the remaining five subsets are used for training. The mean relevance score for each feature is calculated after all the 6 validations are completed.

#### Data Classification Using Support Vector Machines

The features found by the feature selection algorithm were validated by classifying patients into POPCMS and non-POPCMS labels using each of these features individually, as well as collectively. The classification was performed using an exhaustive leave-5-out cross-validation (LOOCV) format using a SVM classifier (Campbell 2002; Shawe-Taylor and Cristianini 2004; Kim 2013). The classification process is explained in (Spiteri et al. 2015). For each combination of test/train subjects in the cross-validation process, the bias point *B* of the SVM classifier is varied between −50 and 50 at increments of 1. This process is repeated 1000 times, and the mean True Positive Rate and False Positive Rate is calculated for each bias point *B*. Each feature chosen by the feature selection process was assessed in this way both individually and collectively with the other chosen features. This process was also applied to the original full set of cerebellar lobule means (grey-level intensity and determinant of Jacobian of deformations separately) for comparison.

### Biomarker Mapping

Each column in each of the two feature matrices (grey-level cerebellar lobule means and determinant of Jacobian of deformations lobule means) represents a different lobule within the cerebellum. A chosen feature therefore corresponds to a single lobule within this area of the brain. Using this information, the resultant biomarkers were mapped back to the labeled cerebellum atlas in order to visualize the lobule’s location on the SUIT template. The lobules location was compared to the SUIT labelled atlas of the cerebellum to gauge a deeper understanding of the significance of these lobules in relation to the development of pediatric POPCMS after surgery.

## Results

### Image Processing

**Fig. 2:**
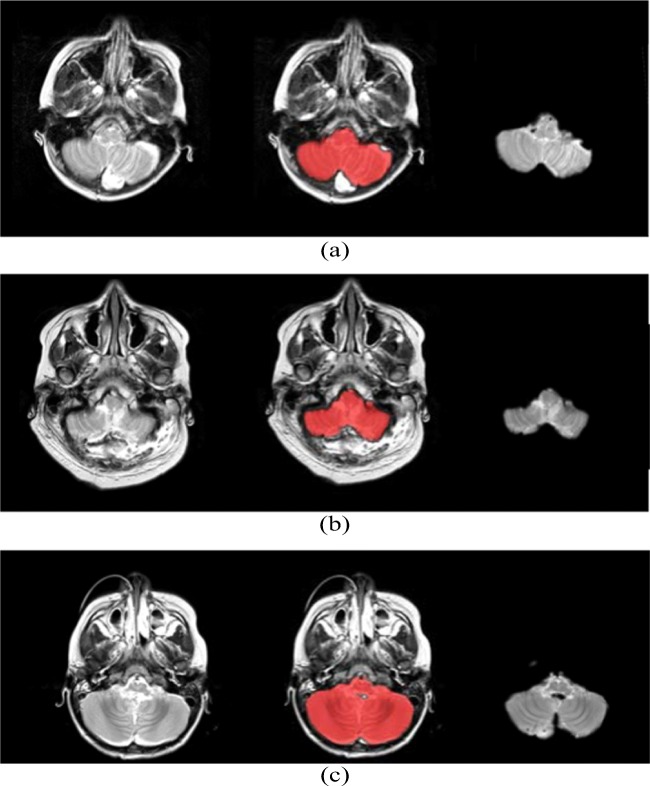
From left to right: original MR image slice, segmentation probability map, and isolated cerebellum on an arbitrary slice from a volumetric MR image for **a** Patient 9 **b** Patient 11 and **c** Patient 13

Figure 2 shows an arbitrary slice from a volumetric brain MR image; the same slice with the cerebellum and brain stem probability map highlighted in red; and the resultant isolated cerebellum. The probability map defines pixels that are likely to form part of the brain stem and cerebellum with a probability exceeding 0*.*5.

### Machine Learning

**Fig. 3:**
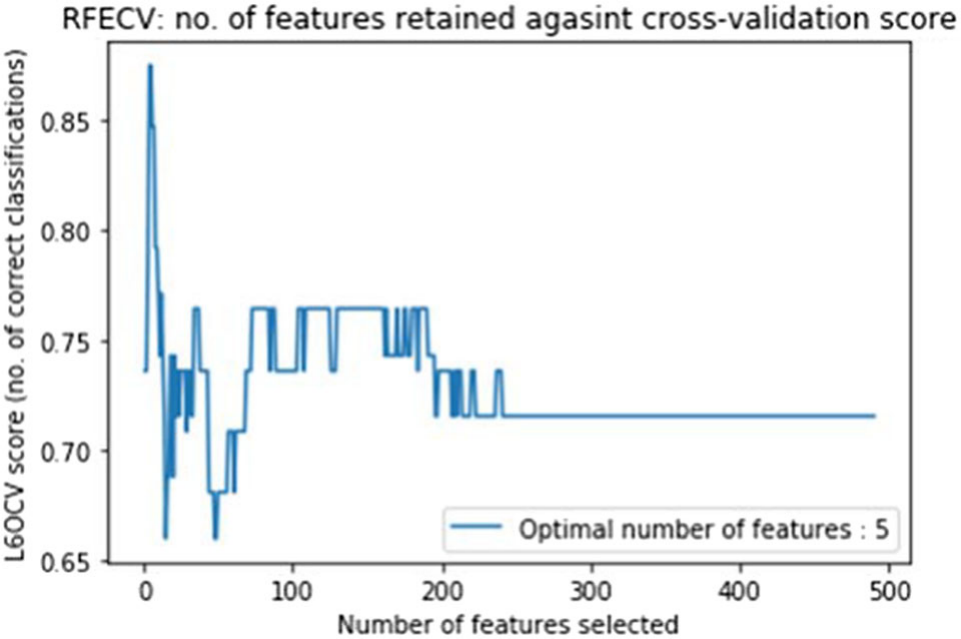
The line graph of features selected (potential biomarkers) against leave-6-out cross-validation classification score obtained using RFECV feature selection algorithm on cerebellar lobule means (grey-level intensity). The optimal number of features is 5

**Fig. 4:**
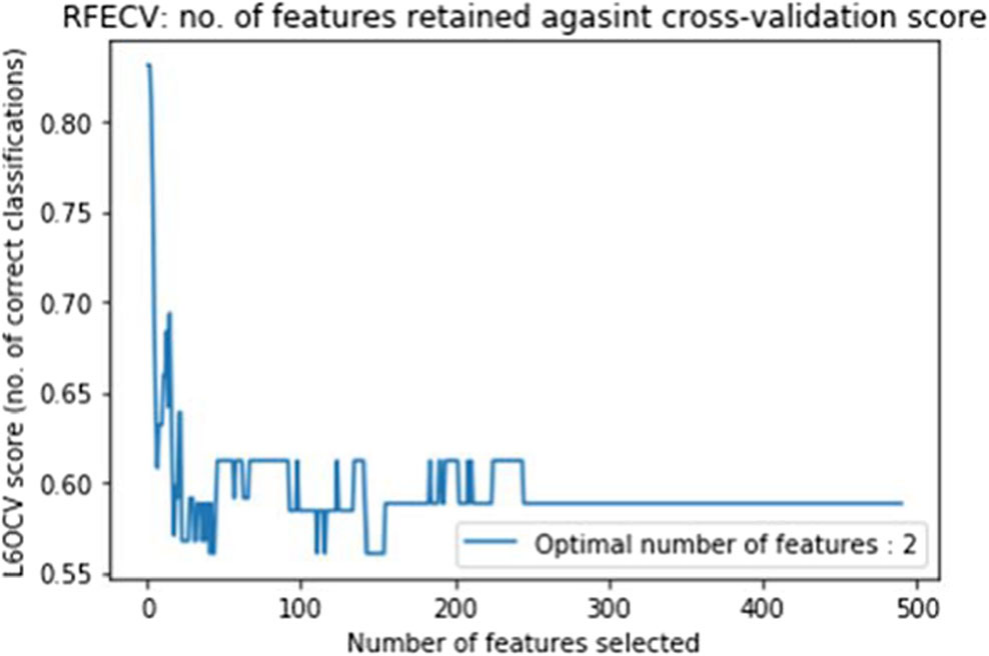
The line graph of features selected (potential biomarkers) against leave-6-out cross-validation classification score obtained using RFECV feature selection algorithm on cerebellar lobule means (determinant of Jacobian of deformations). The optimal number of features is 2

Figures 3 and 4 display the line graphs of potential biomarkers against classification score obtained using the RFECV feature selection algorithm. The classification score is obtained using a leave-6-out cross-validation method applied to the cerebellar lobule means for grey-level intensity (Fig. 3) and determinant of Jacobian of deformations (Fig. 4). In the former, the optimal number of features (potential biomarkers) reported is 5, whilst the optimal number for the latter case is 2.

**Fig. 5:**
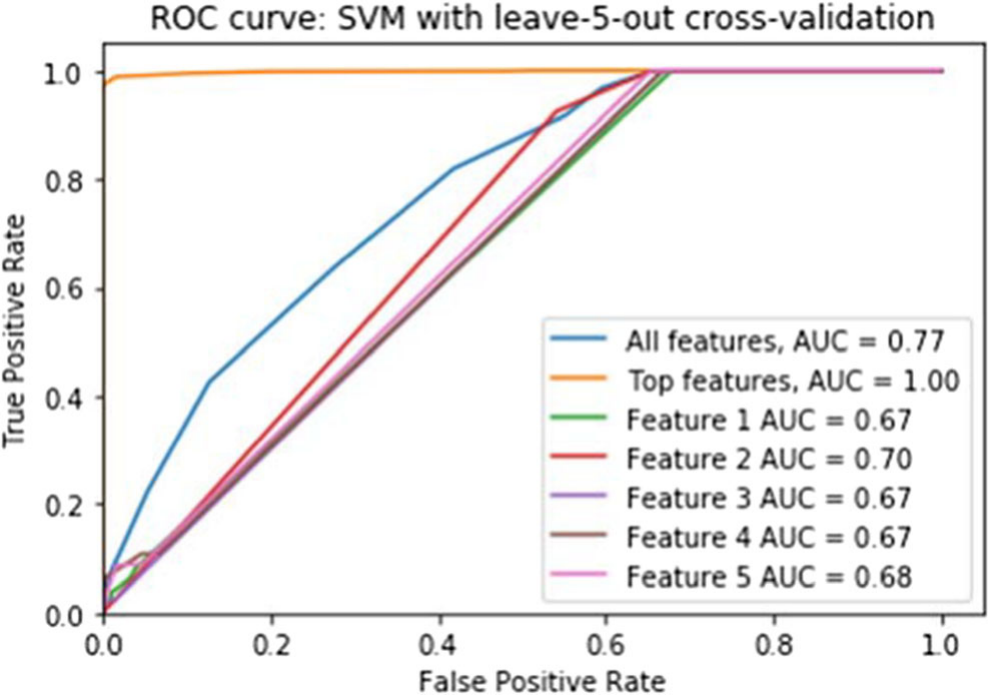
The ROC curves for top-scoring features (potential biomarkers) 1 (month 1), 2 (month 1), 3 (month 1), 4 (month 3), and 5 (month 4) as chosen by the RFECV feature selection algorithm, used to classify subjects as individual features, aggregated as a feature subset, and all the original features (cerebellar lobule means of grey-level intensity)

**Fig. 6:**
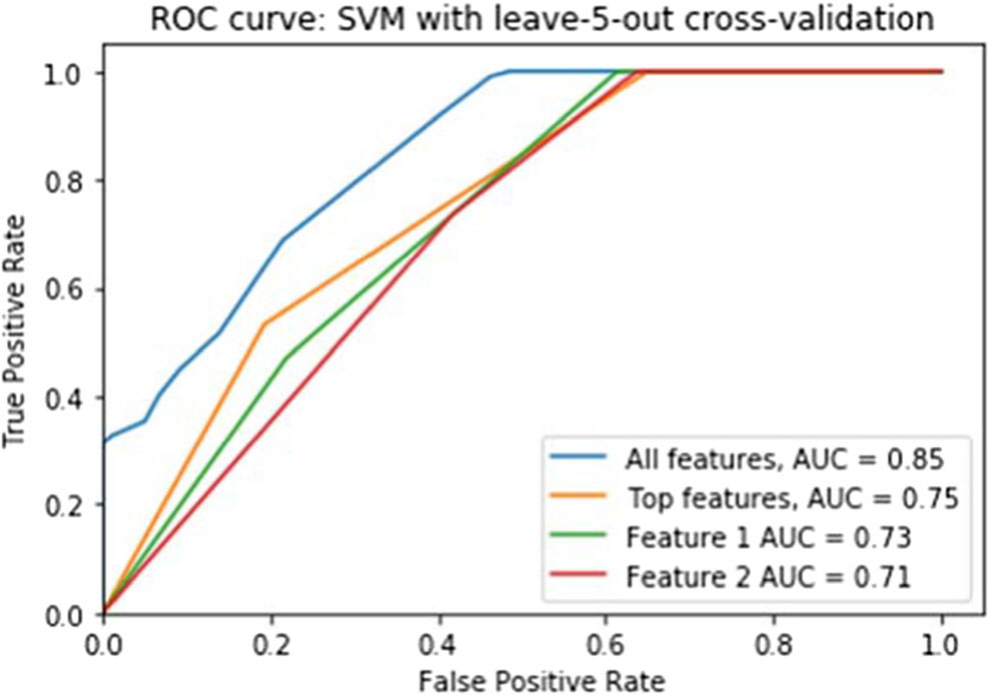
The ROC curves for top-scoring features (potential biomarkers) 1 (month 5) and 2 (month 7) as chosen by the RFECV feature selection algorithm, used to classify subjects as individual features, aggregated as a feature subset, and all the original features (cerebellar lobule means of determinant of Jacobian of deformations)

Figure 5 displays the ROC curves for features (potential biomarkers) 1 (month 1), 2 (month 1), 3 (month 1), 4 (month 3), and 5 (month 4) as chosen by the RFECV feature selection algorithm, used to classify subjects as individual features, aggregated as a feature subset, and all the original features (cerebellar lobule means of grey-level intensity). Figure 6 displays the ROC curves for features (potential biomarkers) 1 (month 5) and 2 (month 7) used to classify subjects as individual features, aggregated as a feature subset, and all the original features (cerebellar lobule means for determinant of Jacobian of deformations).

### Biomarker Mapping

**Fig. 7:**
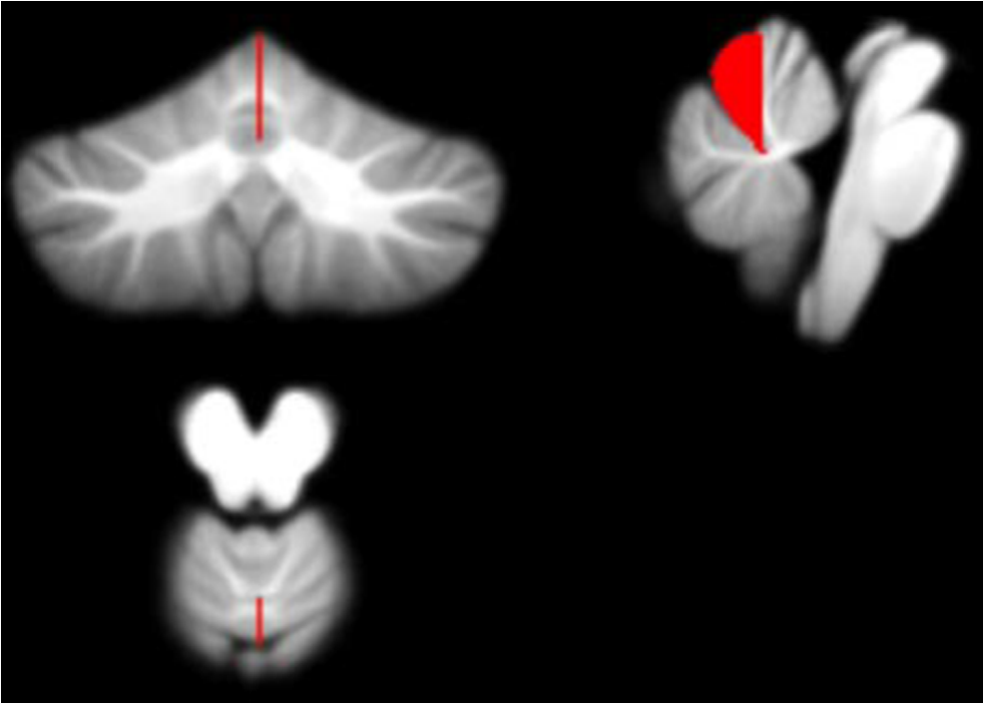
Feature 1 (grey-level intensity) - potential biomarker located in cerebellar lobule Right V (on the SUIT atlas) and identified in Month 1 following surgery, plotted on the SUIT template (red) in the coronal, sagittal, and axial plane (clockwise)

**Fig. 8:**
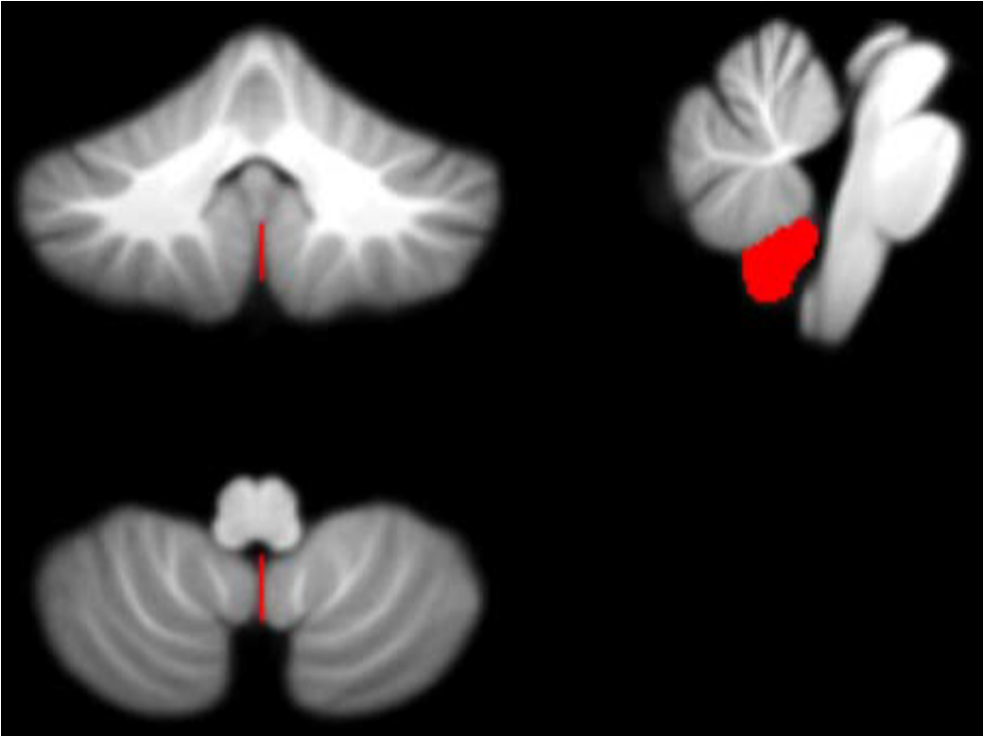
Feature 2 (grey-level intensity) - potential biomarker located in cerebellar lobule Right IX (on the SUIT atlas) and identified in Month 1 following surgery, plotted on the SUIT template (red) in the coronal, sagittal, and axial plane (clockwise)

**Fig. 9:**
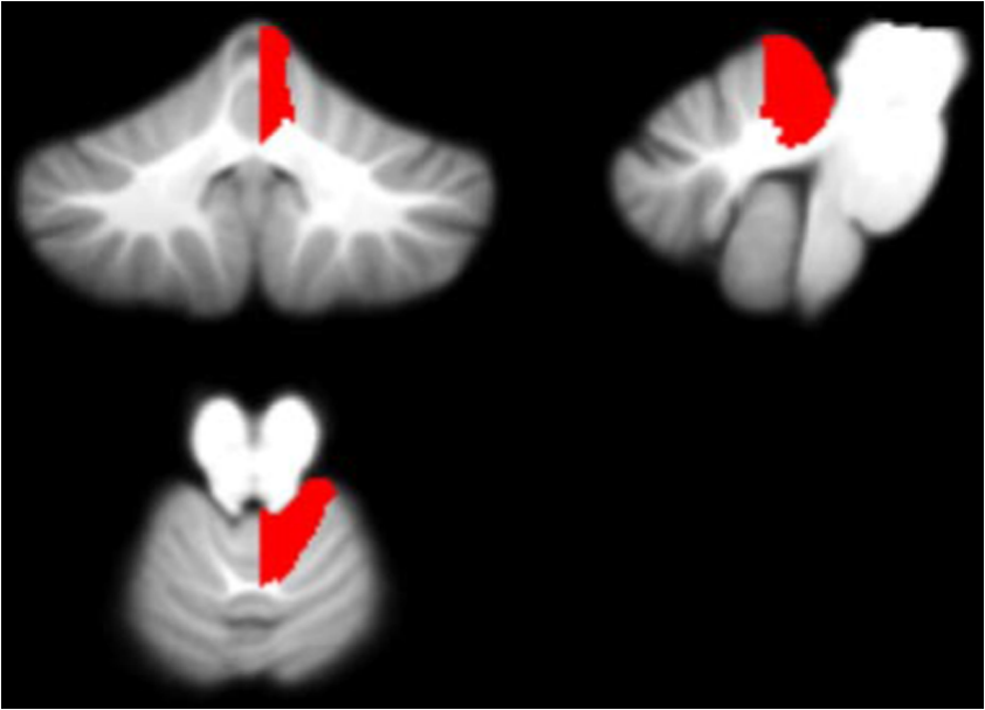
Feature 3 (grey-level intensity) - potential biomarker located in cerebellar lobule Right I-IV (on the SUIT atlas) and identified in Month 1 following surgery, plotted on the SUIT template (red) in the coronal, sagittal, and axial plane (clockwise)

**Fig. 10:**
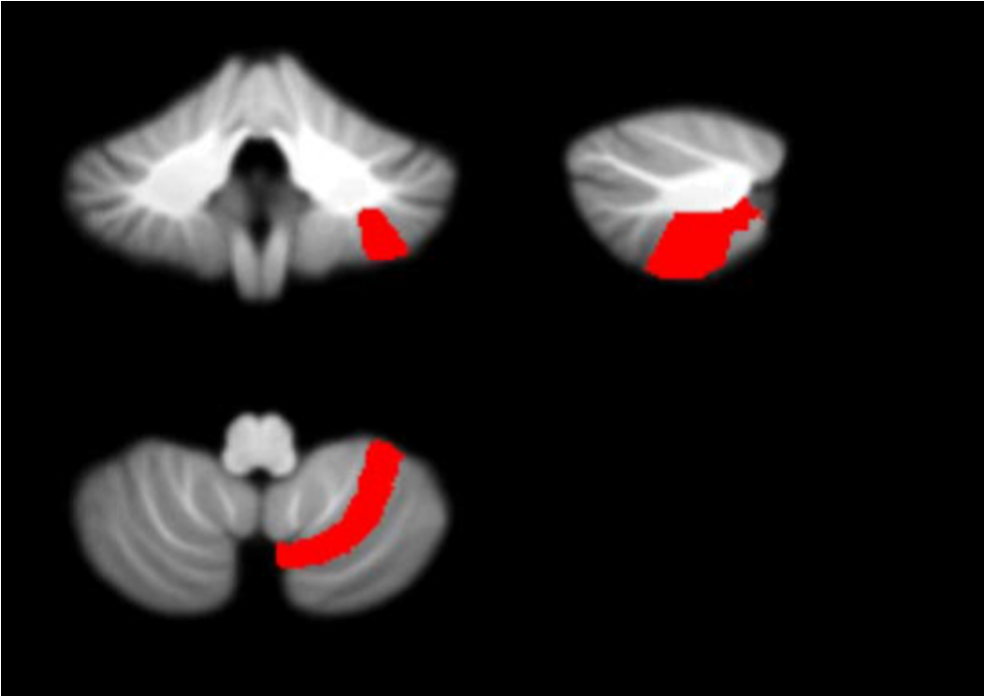
Features 4 and 5 (grey-level intensity) - potential biomarkers located in cerebellar lobule Right VIIIa (on the SUIT atlas) and identified in Months 3 and 4 following surgery plotted on the SUIT template (red) in the coronal, sagittal, and axial plane (clockwise)

Figures 7, 8, 9 and 10 display each of the potential biomarkers which were identified by the RFECV algorithm applied to the cerebellar lobule means of grey-level intensity, mapped onto the SUIT template of the cerebellum and brain stem, in the coronal, sagittal, and axial plane (clockwise). The features displayed correspond to the following potential biomarkers: Feature 1 in the Right V lobule identified in month 1 after surgery; Feature 2 in the Right IX lobule identified in month 1 after surgery; Feature 3 in the Right I-IV lobule identified in month 1 after surgery; Features 4 and 5 in the Right VIIIa lobule identified in months 3 and 4 following surgery.

**Fig. 11:**
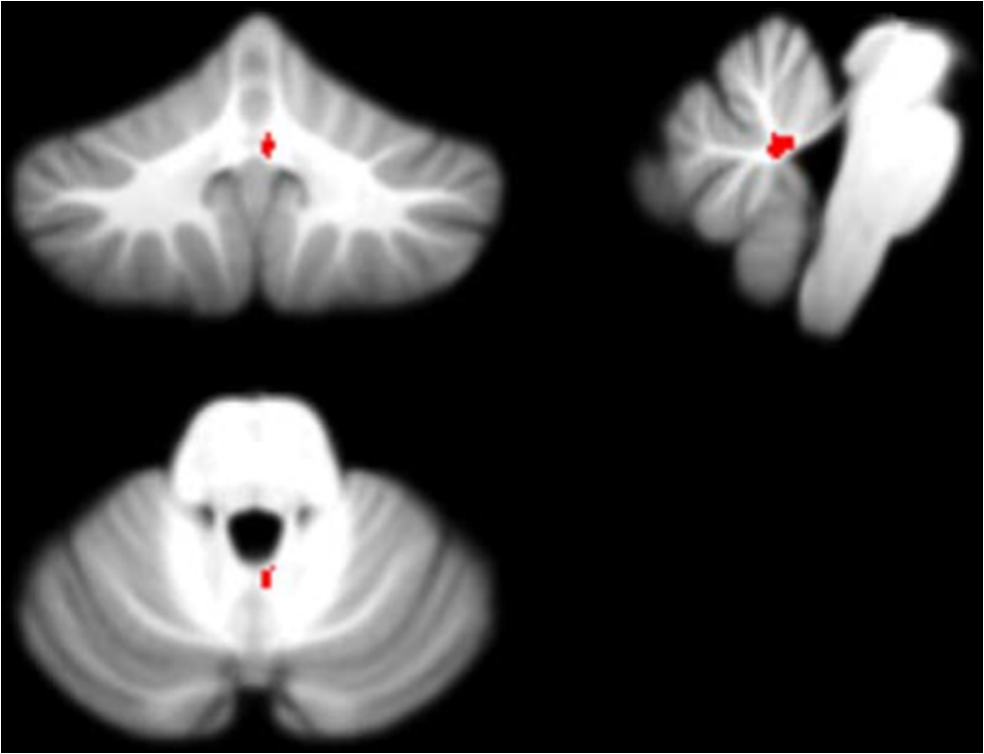
Features 1 and 2 (determinant of Jacobian of deformations) - potential biomarkers located between cerebellar lobules Right I-IV and Vermis (on the SUIT atlas) and identified in Months 5 and 7 following surgery plotted on the SUIT template (red) in the coronal, sagittal, and axial plane (clockwise)

Figure 11 displays the potential biomarkers which were identified for the cerebellar lobule means of determinant of Jacobian of deformations. Both biomarkers where identified within the same lobule occurring in two separate months over the 7-months period. The features displayed correspond to Features 1 and 2 located between the Vermis ad Right I-IV lobule identified in months 5 and 7 following surgery.

The image slices are displayed in neurological convention, such that the left side of the image corresponds to the anatomical left of the subject.

## Computational Variables

The entire post-image processing pipeline took 4.5 min to compute on an Intel(R) Core(TM) i5-8250U CPU at 1.60GHz, 1800 Mhz, 4 Core(s), 8 Logical Processor(s) running Microsoft Windows 10 Pro. This computational time does not include the ROC analysis, the computational time of which is variable and dependent on the number of permutations.

## Discussion

This research presents a novel pipeline for the identification of potential biomarkers for POPCMS from a longitudinal dataset of pediatric brain MRI. The research was split into two main sections, namely image processing and machine learning. The former section included the segmentation and registration of all the images within the dataset to a template of the cerebellum, the computation of the mean grey-level intensity or determinant of Jacobian of deformations within each lobule of the cerebellum, and the longitudinal interpolation of these values over a specified time period to form a feature matrix. The machine learning section consisted of the application of a feature selection algorithm to the feature matrix with the aim of identifying the most salient features that correspond with the occurrence of POPCMS following surgery.

Performing segmentation ensured that the grey-level intensity distribution of each image was normalized across the entire MR imaging dataset. Registering the images back to the template ensured that all the images were in the same coordinate space and therefore aligned. Furthermore, the images were resized to fit the SUIT template and atlas, thus facilitating the automation process of masking each MR image with the labeled SUIT atlas and extracting the cerebellar lobule means. Figure 2 exhibits the results from the segmentation and registration process. It is evident that the SUIT group of functions for spm, together with the SUIT cerebellum atlas is ideal for this analysis and yields robust and accurate segmentation and registration results.

Figures 3 and 4 exhibit the line graph of features selected against the classification score obtained using the RFECV feature selection algorithm for the cerebellar lobule means for grey-level intensity and determinant of Jacobian of deformations interpolated longitudinally over a 7-months period. It is evident that in both cases the peaks of the graph are significantly high at accuracy values between 0.85 and 0.9, and 0.8 and 0.85 for the chosen features subset for grey-level intensity and determinant of Jacobian of deformations, respectively.

The ROC analysis results shown in Fig. 5, corresponding to the optimal feature subset of 5 features chosen for the cerebellar lobule means of grey-level intensity, exhibit a significant increase in area under the curve (AUC) from 0.77 to 1 when only the optimal feature subset of potential biomarkers is sued to classify subjects into POPCMS and non-POPCMS groups. This adds credence to the result displayed in Fig. 2, that these features are in fact plausible candidates for potential biomarkers for this disease. Notwithstanding this, each of the features in isolation scored AUC values of 0.67, 0.7, 0.67, 0.67 and 0.68 respectively for features 1 to 5, therefore each of the individual biomarkers are somewhat reliable at discerning between POPCMS and non-POPCMS subjects when used in isolation.

The ROC analysis results shown in Fig. 6, corresponding to the optimal feature subset of 2 features chosen for the cerebellar lobule means of determinant of Jacobian of deformations did not provide equally successful AUC results. Using features 1 and 2 individually results in AUCs of 0.73 and 0.71, and 0.75 as an aggregated subset. Although these AUC values are both lower than the AUC value of 0.85 for the full feature set, they are still significantly high and the chosen features can still be considered reliable potential biomarkers for this syndrome.

Figures 7, 8, 9, 10 and 11 exhibit the potential biomarkers mapped onto the SUIT template in the coronal, sagittal and axial plane. Considering the first subset of features for grey-level intensity, features 1, 2 and 3 correspond to the Right V, Right IX (adjacent to the vermis lobule IX), and Right I-IV, respectively, and all correspond to month 1 following surgery. These lobules correspond to motor function (Bernard et al. 2012; Buckner et al. 2011; Stoodley and Schmahmann 2009; KH et al. 2012) and language (Bernard and Mittal 2014; Stoodley et al. 2012). Changes in grey-level intensity within the first month following surgery may be related to the symptoms of speech disturbance (Bernard et al. 2012; Buckner et al. 2011; Stoodley and Schmahmann 2009; KH et al. 2012) and decline in motor function (Bernard and Mittal 2014; Stoodley et al. 2012) (for example dysphagia and ataxia) within the first month following surgery of posterior fossa tumors in children with POPCMS (Bernard and Mittal 2014). Features 4 and 5 both fall within the Right VIIIa occurring in months 4 and 5 following surgery. This lobule is also related to the execution of motor function and fine motor control (Bernard et al. 2012; Buckner et al. 2011; Stoodley and Schmahmann 2009; KH et al. 2012; Bernard and Mittal 2014; Stoodley et al. 2012). Furthermore, the lobule Right IX is adjacent to the vermis, which is linked via efferent and afferent fiber tracts to the left inferior olivary nucleus (ION) (Ausim Azizi 2007), which was previously reported as significant in the development of POPCMS following surgery by Spiteri et al. in (Spiteri et al. 2015) Features 1 and 2 for the determinant of Jacobian of deformations correspond to the same area, located between the Vermis and Right I-IV in months 5 and 7 following surgery. This area is located close to the superior cerebellar peduncle (SCP) previously reported as an area of interest in relation to POPCMS in (Avula et al. 2015b). These features add further credence to the features obtained for grey-level intensity as they are located within the same areas responsible for fine motor control (Bernard et al. 2012; Buckner et al. 2011; Stoodley and Schmahmann 2009; KH et al. 2012; Bernard and Mittal 2014; Stoodley et al. 2012).

## Conclusion

The aim of this research was to implement a novel machine learning approach to identify potential biomarkers in 3D space that correlate with the presence of POPCMS, without the bias of prior hypotheses, in a fully automated fashion. A dataset of 40 patients was included in this study.

The registration step allows us to obtain the deformation field that maps the moving image (post-operative MR image) to the fixed image (the SUIT template). Since the registration was split into two steps: affine and non-rigid registration, it was possible to discard the affine matrix that maps the moving image to the fixed image using 12 degrees-of-freedom, and to only analyze the deformation field resulting from the non-rigid part of the deformation. By applying the determinant of Jacobian of deformations, it was possible to quantify shrinkage or enlargement in each area of the cerebellum segment, with respect to the SUIT cerebellum template, across the entire dataset. The cerebellar lobule means of the determinant therefore allows the identification of abnormally enlarged or shrunken lobule within the brain.

The aim was to compare patients over a 7-months period, by comparing each lobule within the volumetric MR image over this time. For each patient, MR images were not acquired within the same frequency post-operatively. This resulted in a sparsely-sampled dataset and therefore missing lobule information for some of the months within the 7-months period, for each patient. The problem of missing MR images was tackled by applying trilinear interpolation.

The results for the feature selection techniques identified 7 potential biomarkers for POPCMS following tumor resection surgery in the posterior fossa. A ROC curve was plotted for each potential biomarker in order to display the trade-off between the true positive rate and false positive rate, as well as the area under the curve (AUC). This gives an indication of the ability of each potential biomarker to discriminate between POPCMS and non-POPCMS subjects.

It is worth noting that all the potential biomarkers identified by this study are located on the right side of the cerebellum. The next step in the study is to correlate the biomarkers with the anatomical findings on the MRI scan to better understand the anatomical substrates involved in the pathophysiology of POPCMS.

MR images in 3D space and the interpolation and in-filling of missing data longitudinally, to provide a full dataset of MR images over a specified time period. In relation to feature extraction from MR images, another contribution includes the use of the determinant of Jacobian of deformations to identify abnormally enlarged or shrunken areas within the brain, in comparison to a template MR image.

This study has identified 7 potential biomarkers in relation to the identification of POPCMS following tumor resection surgery. This is the first study to identify potential biomarkers in the development of this syndrome using machine learning techniques as part of a fully automated pipeline. This pipeline also allows for the identification of important time-points following posterior fossa surgical resection, which will play an important role in understanding the pathophysiology evolution of this condition following surgery.

This novel pipeline can be applied to other datasets or other applications that aim to extract biomarkers for post-operative complications from medical imaging data.

## **Information Sharing Statement**

The SUIT functions used for the segmentation and registration processs, which were developed for spm by Jörn Diedrichsen et al. at the Motor Control and Computational Neuroscience Brain and Mind Institute, Department for Computer Science, University of Western Ontario are available online and explained in detail in (Diedrichsen 2006b). The source code presented in this research paper, together with the anonymized registered cerebella are openly distributed in an online BitcBucket repository. URL:https://bitbucket.org/m_spiteri/popcms-biomarker-identification/src/master/
